# Whole genome mapping as a fast-track tool to assess genomic stability of sequenced *Staphylococcus aureus* strains

**DOI:** 10.1186/1756-0500-7-704

**Published:** 2014-10-08

**Authors:** Julia S Sabirova, Basil Britto Xavier, Margareta Ieven, Herman Goossens, Surbhi Malhotra-Kumar

**Affiliations:** Department of Medical Microbiology, Vaccine & Infectious Disease Institute, Universiteit Antwerpen, Antwerp, Belgium

**Keywords:** *Staphylococcus aureus*, Deletion, ICE*6013*, Whole genome mapping, Whole genome sequencing, USA300

## Abstract

**Background:**

Whole genome (optical) mapping (WGM), a state-of-the-art mapping technology based on the generation of high resolution restriction maps, has so far been used for typing clinical outbreak strains and for mapping *de novo* sequence contigs in genome sequencing projects. We employed WGM to assess the genomic stability of previously sequenced *Staphylococcus aureus* strains that are commonly used in laboratories as reference standards.

**Results:**

*S. aureus* strains (n = 12) were mapped on the Argus™ Optical Mapping System (Opgen Inc, Gaithersburg, USA). Assembly of *NcoI*-restricted DNA molecules, visualization, and editing of whole genome maps was performed employing MapManager and MapSolver softwares (Opgen Inc). *In silico* whole genome *NcoI*-restricted maps were also generated from available sequence data, and compared to the laboratory-generated maps. Strains showing differences between the two maps were resequenced using Nextera XT DNA Sample Preparation Kit and Miseq Reagent Kit V2 (MiSeq, Illumina) and *de novo* assembled into sequence contigs using the Velvet assembly tool. Sequence data were correlated with corresponding whole genome maps to perform contig mapping and genome assembly using MapSolver. Of the twelve strains tested, one (USA300_FPR3757) showed a 19-kbp deletion on WGM compared to its *in silico* generated map and reference sequence data. Resequencing of the USA300_FPR3757 identified the deleted fragment to be a 13kbp-long integrative conjugative element *ICE6013*.

**Conclusions:**

Frequent subculturing and inter-laboratory transfers can induce genomic and therefore, phenotypic changes that could compromise the utility of standard reference strains. WGM can thus be used as a rapid genome screening method to identify genomic rearrangements whose size and type can be confirmed by sequencing.

**Electronic supplementary material:**

The online version of this article (doi:10.1186/1756-0500-7-704) contains supplementary material, which is available to authorized users.

## Background

The plasticity of the bacterial genome is well-known and can be attributed to horizontal gene transfer, genome rearrangements, and the activities of mobile genetic elements (MGEs) [1]. In the case of *Staphylococcus aureus*, and also for several other pathogens, MGE DNA accounts for a substantial part of the identified inter-strain genetic variability in terms of acquisition of virulence, colonization or antibiotic resistance factors. Such MGE-associated changes have also been reported in frozen, archived *S. aureus* strains [2], and are especially problematic for maintenance of reference culture collections utilized for inter- and intra-laboratory experimental validations.

Whole genome (optical) mapping (WGM) is an advanced molecular technology by which endonuclease-digested single DNA molecules are assembled into a high-resolution restriction map constituting a detailed genomic fingerprint. Originally developed in 1993 by Schwartz and colleagues to type *Saccharomyces cerevisiae* strains [3], WGM has so far mainly been applied in clinical microbiology for typing outbreak strains [4–7], and for genome sequencing to guide *de novo* assembly of sequence contigs [8]. Here, we applied WGM to rapidly assess genomic stability of key reference strains of *Staphylococcus aureus* utilizing their previously published whole genome sequences as comparators.

## Results and discussion

**Figure 1:**
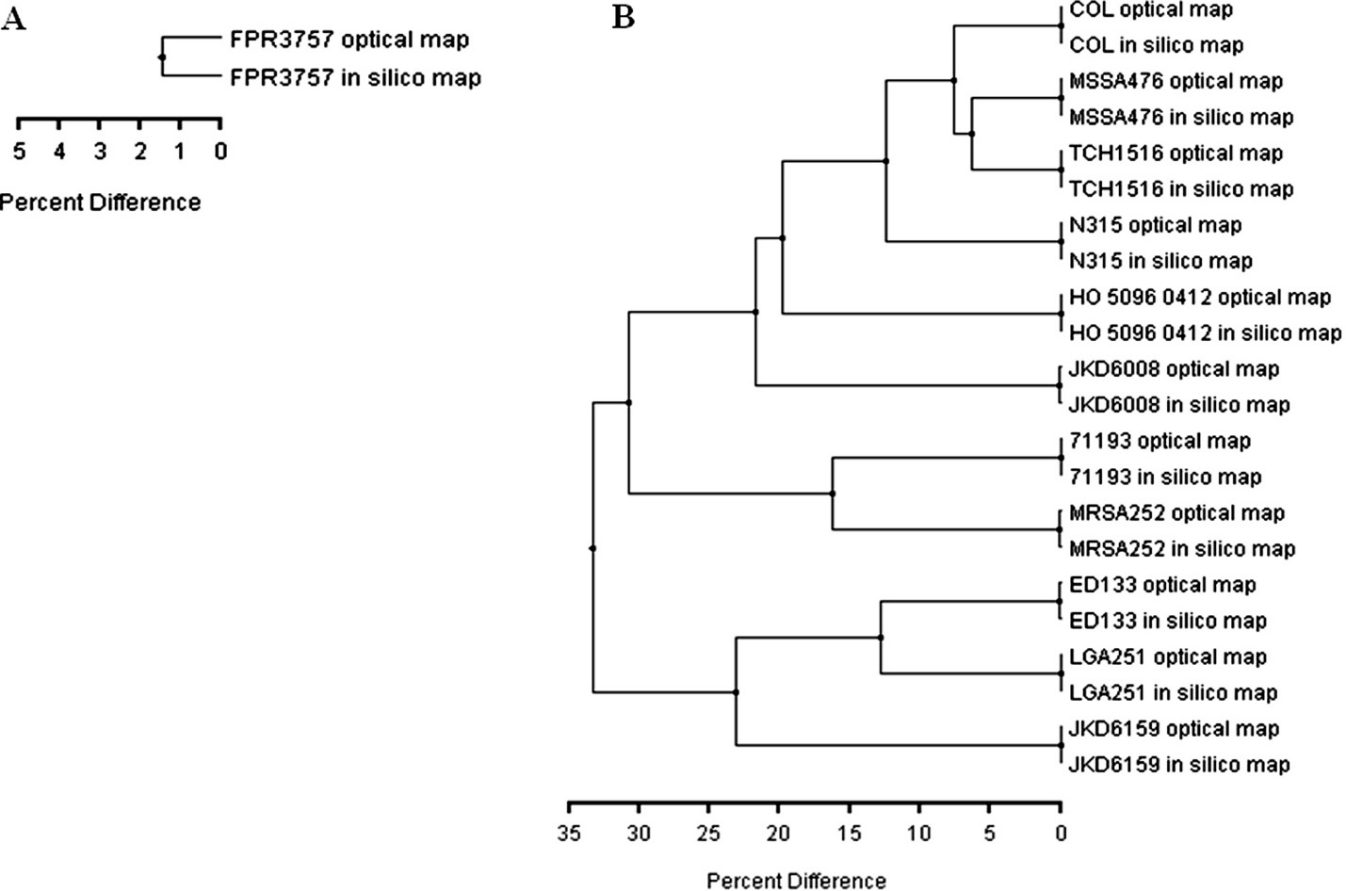
**Map similarity cluster of experimental whole genome maps and corresponding** ***in silico*** **maps for** ***S. aureus*** **strains constructed using unweighted-pair group method with arithmetic averages (UPGMA).** Whole genome map of FPR3757 features 1.5% map distance **(A)**, whereas whole genome maps of other strains feature 0–0.1% map distance compared to their corresponding *in silico* maps **(B)**.

**Figure 2:**
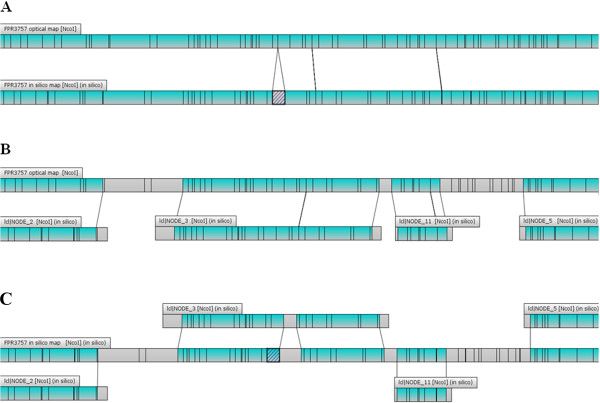
**Aligned experimental and** ***in silico*** **whole genome maps of FPR3757 (A); alignment of experimental map of FPR3757 to sequence contigs (B); alignment of** ***in silico*** **map of FPR3757 to sequence contigs (C).** Regions highlighted in green indicate perfect alignment. The region corresponding to the deletion is colored in grey and hashed with dark grey diagonal bars in the *in silico* map.

**Figure 3:**
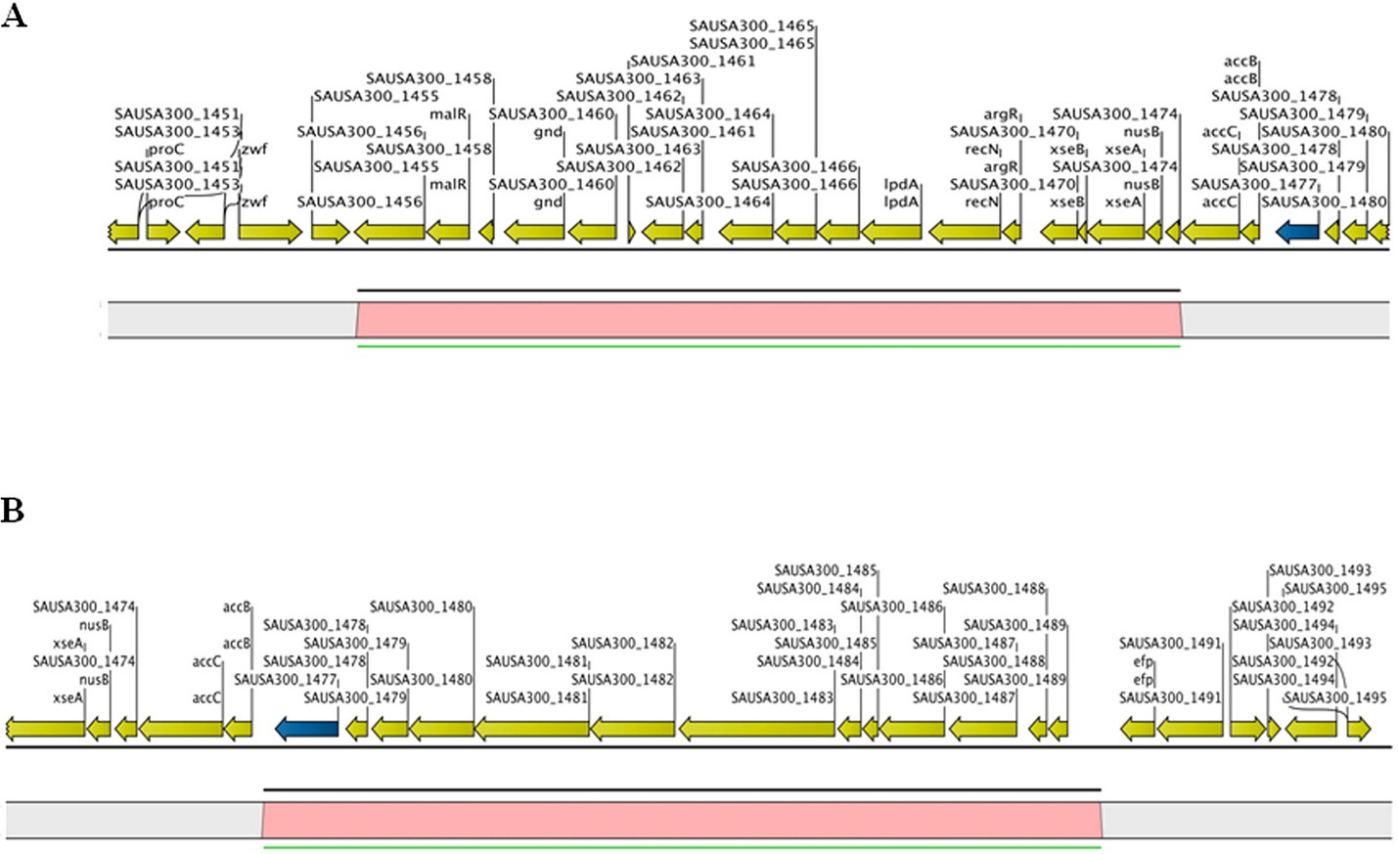
**Genomic deletion in FPR3757 according to WGM (A), and whole genome (re) sequencing (B).** All open reading frames are highlighted in yellow, transposase gene is highlighted in blue. Region corresponding to the genomic deletion is highlighted in pink.

We utilized WGM to assess the genomic stability of previously sequenced *S. aureus* that are also commonly used as laboratory reference strains (Additional file 1: Table S1). Comparison of experimental whole genome maps to *in silico* restriction maps (*NcoI*) generated from published sequences showed that for majority of the strains (COL, MRSA476, 71193, ED133, JKD6008, JKD6159, LGA251, MRSA252, N315, TCH1516, and HO 5096 0412), both maps were identical (100–99.9% map similarity; Figure 1). The 0.1% difference in *in silico* and experimental maps of some strains (COL, MRSA476, 71193, ED133, JKD6008, LGA251, MRSA252, N315, and TCH1516) could be attributed to the loss of small DNA fragments in the experimental maps. The rate of loss of fragments ≤ 2 kb could be as high as 75% during mapcard processing comprising *in situ* restriction, staining and washing of linearized and immobilized DNA molecules, as described previously [9]. However, for *S. aureus* FPR3757, a clear 1.5% map difference was observed between the experimental and corresponding *in silico* WGMs. The experimental WGM of FPR3757 displayed a 19-kbp genomic deletion compared to its *in silico* map (Figure 2). According to published sequence data, the deleted fragment contained genes SAUSA300_1456 to SAUSA300_1474 that included a maltose degradation operon (Figure 3A).

In order to confirm the genomic deletion detected by WGM, the entire genome of FPR3757 was sequenced, *de novo* assembled into sequence contigs by Velvet with an optimized *k*-mer [10] and compared to its corresponding published genome using MapSolver. Re-sequencing of FPR3757 showed that the deleted fragment was 13354 bp long (nucleotides 1630722 and 1644075) and comprised genes SAUSA300_1477 to SAUSA300_1489 (Figure 3B). The observed difference in prediction of the site of deletion by WGM and sequencing is likely to be due to the loss of small fragments during preparation of whole genome maps. Indeed, upon inspection of the FPR3757 map, the total number of restriction fragments was found to be remarkably lower than in the *in silico* map (219 and 244 fragments, respectively). Loss of fragments shorter than 2 kb can result in a certain degree of imprecision in prediction of the size of restriction fragments and localization of *NcoI* sites in the whole genome map, as compared to the *in silico*-generated map.

**Figure 4:**
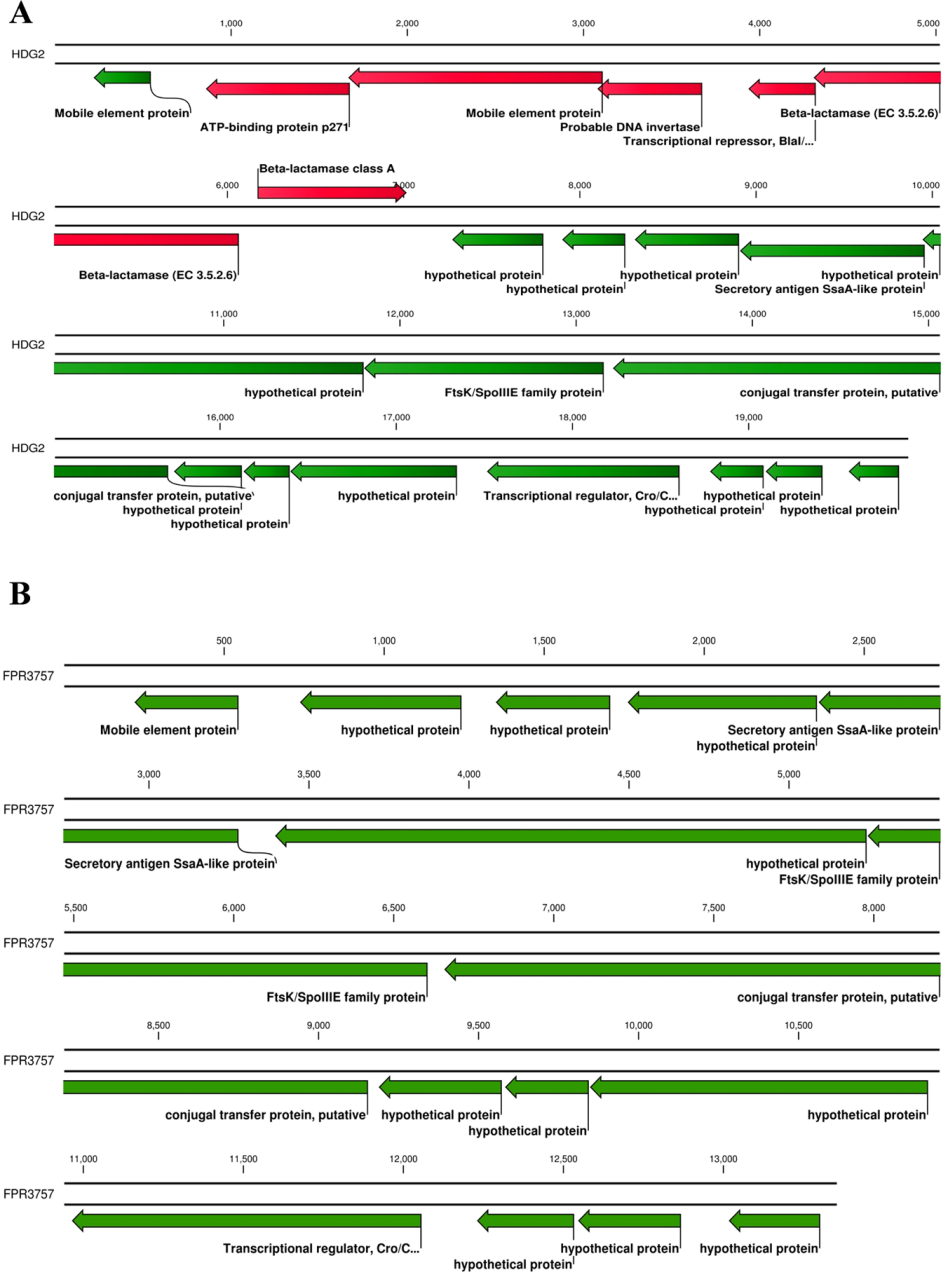
**Detailed view of** ***ICE6053*** **of** ***S. aureus*** **strain HDG2 (A) and of** ***ICE6053*** **of** ***S. aureus*** **strain FPR3757 (B).** Gene annotation was performed using RAST annotation server and blast2go. Genes highlighted in red in HDG2 represent those deleted in FPR3757.

BLAST searches against *S. aureus* published genomes using the nucleotide sequence of the deleted fragment as a query identified an integrative conjugative element *ICE6013*, which was recently described as a novel MGE in a number of MRSA [11]. The MGE in FPR3757 showed 99% pairwise identity to the *ICE6053* of *S. aureus* strain HDG2 (accession number FJ231270), except for the absence of a 6551-bp *Tn552* transposon. Comparison of the two ICEs employing blast2go and RAST functional comparison tools allowed to assign putative functions to the genes and to compare their genetic contents: the absence of *Tn552* in the ICE in FPR3757 resulted in the loss of a β-lactamase operon comprising *blaI, blaR1* and *blaZ* genes, whereas other genes typical for ICEs and potentially responsible for their transposition, replication and conjugative transfer were found to be intact [12, 13] (Figure 4, Additional file 2: Table S2).

## Conclusions

In conclusion, WGM of sequenced *S. aureus* strains detected a large genomic deletion in one out of twelve strains studied here. Subsequent genome sequencing of the altered genome and inspection of the region identified the exact size, genomic location and the nature of this genomic deletion. WGM can thus be employed to rapidly assess genomic stability of strains over time during storage or after inter-laboratory transport followed by corroboration of genomic changes by whole genome sequencing.

## Methods

### Bacterial strains and growth conditions

Twelve *S. aureus* strains that are commonly used as reference strains and whose whole genome sequences are available in public databases were requested from strain collections (Network on Antimicrobial Resistance in *S. aureus* (NARSA, http://www.beiresources.org/), or from research groups: COL (Acc.no. NC_002951), 71193 (Acc.no. NC_017673), FPR3757 (Acc.no. NC_007793), ED133 (Acc.no. NC_017337), JKD6008 (Acc.no. NC_017341), JKD6159 (Acc.no. NC_017338), LGA251 (Acc.no. NC_017349), MRSA252 (Acc.no. NC_002952), MRSA476 (Acc.no. NC_002953), N315 (Acc.no. NC_002745), TCH1516 (Acc.no. NC_010079), and HO 5096 0412 (Acc.no. NC_017763). Upon arrival, bacterial stocks were inoculated on blood agar plates and incubated overnight at 37°C. 16 hour-old colonies were used for species re-confirmation by Maldi-TOF and to prepare glycerol stocks for long-term storage at -80°C. In parallel, one of the 16 hour-old colonies was inoculated on brain heart infusion agar, incubated overnight at 37°C and used for WGM.

### Genome sequencing

*S. aureus* strains were grown on brain heart infusion plates for 24 h at 37°C and used to extract genomic DNA using MasterPure™ Gram Positive DNA Purification Kit (Epicentre Technologies Corp). Samples were prepared and sequenced employing Nextera XT DNA Sample Preparation Kit and Miseq Reagent Kit V2 (Illumina Inc). The 2 X 150 reads were *de novo* assembled into contigs using CLC Genomics Workbench with default parameters and Velvet v1.2.10 assembler [14] with an optimised N50 *k-mer* size of 93 [10]. Sequencing data were correlated with corresponding whole genome maps to perform contig mapping in MapSolver software (Opgen Inc). Gene annotation of ICE elements was performed using RAST annotation server and blast2go for functional annotation.

### Whole genome mapping

Maps were generated on an Argus™ Optical Mapping System (Opgen Inc, Gaithersburg, USA). DNA extraction, quality control, restriction using *NcoI*, and loading on a mapcard were done according to manufacturer's protocols. Briefly, *S. aureus* colonies grown overnight at 37°C on brain heart infusion plates were used for isolation of high molecular weight (HMW) DNA using Argus® HMW DNA isolation kit (Opgen, Inc). Extracted DNA preps were checked for the presence of HMW DNA molecules by using Argus® QCard kit (Opgen, Inc) and subsequently used for mapping employing Argus® MapCard II kit (Opgen, Inc). The assembly of restricted DNA molecules and identification of novel *NcoI* restriction sites was performed employing MapManager software (Opgen Inc.). Visualization and editing of maps was performed using MapSolver software (Opgen Inc.). For map editing, whole genome maps were adjusted in size, orientation and in starting point employing *in silico* maps generated from corresponding genome sequence data using MapSolver’s function “Comparative genomics” and an advanced parameter “Allow alignments to wrap circular maps” with other default parameters pre-set in MapSolver. Map clustering was also performed using MapSolver’s default parameters employing MapSolver’s function “Create cluster”.

## Electronic supplementary material

Additional file 1: Table S1: *Staphylococcus aureus* strains used in this study. (DOCX 17 KB)

Additional file 2: Table S2: List of genes predicted in ICE6053 of *S. aureus* strain FPR3757 by RAST annotation server and blast2go. (DOCX 16 KB)

## Abbreviations

WGM: Whole genome mapping
ICE: Integrative conjugative element
MGE: Mobile genetic element
BLAST: Basic local alignment search tool
NARSA: Network on Antimicrobial Resistance in *S. aureus.*
