# Supplementary material for: Whole genome mapping as a fast-track tool to assess genomic stability of sequenced Staphylococcus aureus strains
Source: BMC Res Notes. 2014 Oct 8;7:704. doi: 10.1186/1756-0500-7-704 (PMC4197248; doi:10.1186/1756-0500-7-704)
Supplement: Supplementary file 1 — Additional file 1: Table S1: Staphylococcus aureus strains used in this study. (DOCX 17 KB) [file 13104_2014_3228_MOESM1_ESM.docx]

**Table S1.** *Staphylococcus aureus* strains used in this study.

| **Name** | **Acronym** | **Characteristics** | **Accession number** | **Submission Date** |
| --- | --- | --- | --- | --- |
| *Staphylococcus aureus* subsp. aureus COL | COL | Human MRSA | NC_002951 | 10-Jun-2013 |
| *Staphylococcus aureus* subsp. aureus 71193 | 71193 | Livestock-associated MRSA | NC_017673 | 27-Aug-2013 |
| *Staphylococcus aureus* subsp. aureus USA300_FPR3757 | FPR3757 | Community acquired MRSA | NC_007793 | 10-Jun-2013 |
| *Staphylococcus aureus* subsp. aureus ED133 | ED133 | Ovine strain Ovine/mastitis | NC_017337 | 28-Aug-2013 |
| *Staphylococcus aureus* subsp. aureus str. JKD6008 | JKD6008 | Hospital acquired MRSA | NC_017341 | 27-Aug-2013 |
| *Staphylococcus aureus* subsp. aureus JKD6159 | JKD6159 | Community acquired MRSA | NC_017338 | 28-Aug-2013 |
| *Staphylococcus aureus* subsp. aureus LGA251 | LGA251 | Livestock-associated MRSA | NC_017349 | 11-Jun-2013 |
| *Staphylococcus aureus* subsp. aureus strain MRSA252 | MRSA252 | Hospital acquired MRSA | NC_002952 | 10-Jun-2013 |
| *Staphylococcus aureus* strain MSSA476 | MSSA476 | Community-acquired MSSA | NC_002953 | 10-Jun-2013 |
| *Staphylococcus aureus* subsp. aureus N315 | N315 | Human MRSA | NC_002745 | 26-Aug-2013 |
| *Staphylococcus aureus* subsp. aureus USA300_TCH1516 | TCH1516 | Community acquired MRSA | NC_010079 | 27-Aug-2013 |
| *Staphylococcus aureus* subsp. aureus HO 5096 0412 | HO 5096 0412 | MRSA | NC_017763 | 11-Jun-2013 |
