# Supplementary material for: Whole genome mapping as a fast-track tool to assess genomic stability of sequenced Staphylococcus aureus strains
Source: BMC Res Notes. 2014 Oct 8;7:704. doi: 10.1186/1756-0500-7-704 (PMC4197248; doi:10.1186/1756-0500-7-704)
Supplement: Supplementary file 2 — Additional file 2: Table S2: List of genes predicted in ICE6053 of S. aureus strain FPR3757 by RAST annotation server and blast2go. (DOCX 16 KB) [file 13104_2014_3228_MOESM2_ESM.docx]

**Table S2.** ORFs predicted in ICE6053 of *S. aureus* strain FPR3757 by blast2go and RAST functional comparison tools.

| ORF number | Start | Stop | Strand | Annotation |
| --- | --- | --- | --- | --- |
| ORF1 | 544 | 221 | - | Mobile element protein |
| ORF2 | 1241 | 738 | - | Hypothetical protein |
| ORF3 | 1706 | 1350 | - | Hypothetical protein |
| ORF4 | 2352 | 1762 | - | Hypothetical protein |
| ORF5 | 3279 | 2359 | - | Secretory antigen SsaA-like protein |
| ORF6 | 5242 | 3395 | - | Membrane protein, putative |
| ORF7 | 6605 | 5247 | - | FtsK/SpoIIIE family protein |
| ORF8 | 9154 | 6659 | - | Conjugal transfer protein, putative |
| ORF9 | 9572 | 9189 | - | Hypothetical protein |
| ORF10 | 9844 | 9584 | - | Hypothetical protein |
| ORF11 | 10904 | 9849 | - | Hypothetical protein |
| ORF12 | 12056 | 10965 | - | Transcriptional regulator, Cro/CI family |
| ORF13 | 12533 | 12231 | - | Hypothetical protein |
| ORF14 | 12867 | 12547 | - | Hypothetical protein |
| ORF15 | 13302 | 13018 | - | Hypothetical protein |
